# Protection of muscle nuclei

**DOI:** 10.18632/oncotarget.5240

**Published:** 2015-08-22

**Authors:** Talila Volk, Shuoshuo Wang

**Affiliations:** Department of Molecular Genetics, Weizmann Institute of Science, Rehovot, Israel

**Keywords:** nucleus, lamin, muscle, HP1, nuclear biomechanics

Nuclear morphology and architecture are maintained by nucleoskeletal elements including, chromatin associated factors and nuclear lamina components both of which are involved in the regulation of chromatin 3D organization [1, 2]. As such, differentiated cells maintain a tissue-specific nuclear distribution of the eu- and heterochromatin and contain steady-state levels of nuclear lamina components and chromatin factors. The mechanical forces applied to the cell nuclei in a given tissue provide a mechano-sensitive signal, which is tightly linked to the levels of the nuclear lamina component lamin A/C [3]. Since lamin A/C associates and regulates the activity of nuclear factors including chromatin regulators and transcription factors it may facilitate the interpretation of the mechanical signal from the cytoplasm into the nucleus.

In striated muscle fibers the nuclei are exposed to variable mechanical forces resulting from contraction/relaxation waves. Surprisingly, their nuclear shape and position are invariably robust. Notably, numerous muscle diseases exhibit abnormal nuclear morphology and nuclear aggregation phenotypes suggesting a link between the aberrant nuclear morphology and defective muscle function. However, direct evidence is required to elucidate this possible link.

The Linker of Nucleoskeleton and Cytoplasm (LINC) complex functions as the major nuclear force transmitting machinery in eukaryotic cells [4, 5]. It comprises three protein families: (1) Nesprins, which are large proteins that contain an evolutionary conserved KASH domain inserted into the outer nuclear membrane, multiple spectrin repeats domain, and an N-terminal domain that associates with various cytoskeletal elements (i.e., F-actin, microtubules, or intermediate filaments), (2) trimeric SUN proteins, which contain a C-terminal SUN domain inserted into the inner nuclear membrane associating directly with triple KASH domain proteins at the perinuclear space, and (3) lamins A/C, and B, which consist the inner nuclear membrane and associate with various chromatin and transcriptional regulators. Different cell types contain different combinations of the LINC complex proteins. Their knock out phenotypes are often pleiotropic, affecting mainly skeletal and cardiac muscles, presumably reflecting the need to protect nuclear shape in tissues with altered cytoplasmic strain.

In our study we used the *Drosophila* larval muscles, which similarly to vertebrate muscles, are striated and multinucleated, to reveal the molecular basis and biological significance of the robust shape and position of the myonuclei.

Previously, we reported a significant disruption of nuclear morphology and position in muscle fibers from homozygous mutant larvae lacking functional MSP300/Nesprin or Klar, representing the only KASH-containing proteins in *Drosophila* [6]. Notably, in both mutants muscle function was severely impaired. In the present manuscript, we identify two additional components required for maintaining uniform nuclear morphology. Both components, namely ACF-7/Shot, and EB1, are microtubule-associated proteins observed specifically at the nuclear membrane. Functionally, these proteins interact with MSP300/Nesprin to organize a specialized perinuclear microtubule network linked to the nuclear membrane. If any of these components is missing nuclear morphology is severely impaired similarly to mutants of the *MSP300/Nesprin* and *klar* [7].

**Figure 1:**
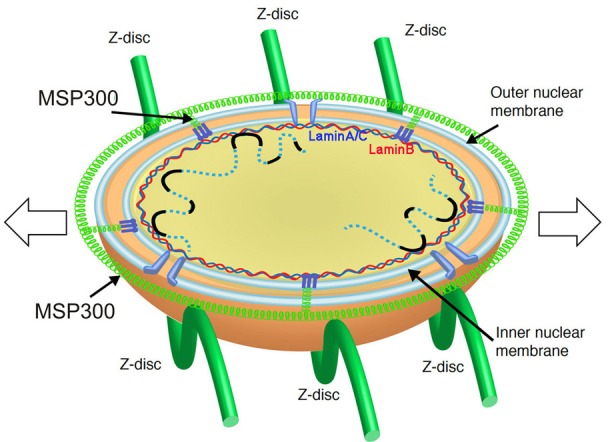
A model describing the components essential for maintaining homogenous myonuclear shape A schematic model describing the lower half of the myonucleus facing the sarcomeric myofilaments. It is surrounded by MSP300, which provides elastic shield that protects the nucleus. The heterochromatin organization is maintained through its association with the nuclear lamina, which associates with the inner nuclear membrane.

It has been predicted that in order to absorb and antagonize the variable mechanical forces applied to the muscle nuclei during muscle contraction/relaxation waves, the LINC complex should provide the myonuclei with both a rigid shield and elastic properties. We found that the perinuclear microtubules provide the rigid nuclear shield, because in muscle-specific knock down of the microtubule associated proteins Shot/ACF7 and EB1 nuclear morphology was significantly impaired. To identify the flexible component we applied external forces on the muscles by stretching the larva. Whereas the perinuclear microtubule network did not change a nucleus-associated ring composed of “KASH-less” isoforms of Nesprin/MSP300 was expanded in response to muscle stretching. Analysis of MSP300 distribution using the anti MSP300 antibody, which is reactive with the spectrin repeats region, revealed small dots representing the epitope. Notably, the distance between these dots increased significantly in response to muscle stretching. This observation, together with previous molecular dynamic data used to demonstrate the flexible properties of the multiple spectrin repeats, led us to conclude that Nesprin/MSP300 provides elastic properties to the myonuclei, thus enabling their accommodation to the variable cytoplasmic strain (Figure 1).

To reveal the functional significance of maintaining a robust and homogenous nuclear morphology, we analyzed the levels of the nuclear lamina components lamin A/C and B, as well as the level of heterochromatin Protein 1 (HP1), in *MSP300/Nesprin* and *klar* mutants. Strikingly, we found that the levels of lamin A/C and HP1 were significantly reduced and substantial changes in their protein distribution were observed in these mutants. Nevertheless, only subtle changes in lamin B levels were observed. Both lamin A/C and HP1 were shown to regulate gene expression levels in other systems Therefore, we postulate that the changes in nuclear morphology observed in mutants of the LINC complex lead to profound changes in gene expression levels in the myonuclei. This effect may explain the severe muscle abnormalities detected in patients suffering from mutations in these genes.

We conclude that the LINC with its associated protein complex is essential for protecting and maintaining integral myonuclei morphology. It is predicted that a major consequence of mutations affecting proteins of this complex would be disruption of the transcriptional output affecting the characteristic protein repertoire of the cell, which is essential for muscle function and particularly for muscle response to external stimuli.
